# Our Weaker Brethren
*The Feeble-Minded Child and Adult: A Report on an Investigation of the Physical and Mental Condition of 50,000 School Children, with suggestions for the better Education and Care of the Feeble-minded Children and Adults." (London: Swan Sonnenschein.)


**Published:** 1893-07-08

**Authors:** 


### Our Weaker Brethren.*

Between the imbecile and the dullest of the sane comes a numerous class, partaking of the characteristics of both, deprived of the privileges of either. There are asylums for the insane and for idiots, complete helplessness claims and receives protection; but what is done for those who, if properly supervised, are not helpless, but who are totally unfit to guide their own steps through the mazes of this world? Practically nothing. Send them to an asylum, and they are sent back as "not suitable"; put them to work under ordinary circumstances, and they are dismissed as unreliable. They drift to the prison or the workhouse, places where a weak judgment is most likely to be swayed to evil, even if misfortune more than crime has led them to these shelters first, and whereas they might have become, with proper care, modestly useful in a feeble way, they develop into something absolutely noxious.

The Charity Organization Society has published a number of statistics taken from the study of 50,000 school children. We cannot quite accept these at the compiler's valuation. The physical signs on which he largely relied seem to us vague and misleading, and he explains that their importance is decided largely by their "conjunction," a phrase which smacks too much of the phrenologist and the astrologer. Let a stranger enter a schoolroom and ask a hundred children to look him in the face and hold out their hands, and others than those who are wanting in intellect will obey the order with some sign of eccentricity that will be held to condemn them. Perhaps the vagueness lies in the description of the method rather than in the method itself, but we should like to see it in operation before we accept it as satisfactory.

Allowing, however, for all possible numerical inaccuracy, we have the serious fact that there are among us a very considerable number of people who are just short of imbecility. They belong to all classes, and to the well-to-do in larger proportion than the poor. The reason of this is obvious enough. Medical skill, good food, careful tendance preserve many lives among the rich, which in poor homes would be snuffed out in the struggle for existence where bread is scant and air is scantier still. But from the point of view of practical charity these cases do not concern us. Their parents or kinsfolk provide for them. It is the weak-minded children of the poor who form a real anxiety.

One reason for this is clear enough when we say that it is estimated that out of a total of 550 imbeciles, 155, equivalent to 28 per cent., were predisposed by heredity. The details of these cases are as follow. The predisposition existed in :—

21 cases in the father.
37 ,, ,, mother.
25 ,, in both parents.
52 ,, in the family of father or mother.
20 ,, in brothers and sisters.

These statistics are taken from cases in Denmark, but the state of matters implied in them exists in England also. Men and women of feeble mind marry and become parents. This cannot be avoided as our law now stands. These people are not insane; they understand what marriage means, but they are fundamentally unfit for its responsibilities. Some straitening of the law might prevent marriage in the case of those who are themselves of weak mind—though at the risk of the veto being abused at the instance of unscrupulous kindred who might produce any deviation from the commonplace as an instance of mental abnormality—but with regard to those who are themselves *compos mentis*, but have feeble-minded relations, nothing can be done except by raising the standard of the public conscience. And here again we are met with a difficulty. For while a family predisposition exists in many cases, there are others where the weakness, amounting even to idiocy, of a brother, a sister, or a relative, implies no hereditary taint; for of 2,380 cases investigated by Drs. Shuttleworth and Fletcher Beach, no fewer than 43 per cent. were due to difficulties or accidents of birth, and 29·87 to abnormal conditions of the mother before her child's birth —conditions which obviously might exist in the case of one member of a family and in that of no other. Again, infantile convulsions, responsible for 27·39 per cent., cannot be regarded as a family disease. When we come to imbecile, epileptic, or neurotic taints in the health record, to which are due 41·38 per cent. of cases, or to the phthisical tendency, which marks 28·31, things are different; but how difficult it is to probe back to first causes. Contrary to general belief, consanguineous marriages and even parental intemperance seem to have no very great influence on feebleness of mind. The first are responsible for only 4·20 per cent.; the latter is the cause attributed in 16·38.

It is, of course, simply a matter of humanity to extend to the feeble minded the kindness and protection we show to the more severely afflicted of our kind; but it develops into a question of morality and public welfare when we consider all the circumstances of the case. It is, in some aspects, specially a woman's question, and as such it has engaged the attention of the Metropolitan Association for Befriending Young Servants, the Young Women's Christian Association, and similar societies. Feeble-minded girls, not imbecile, but lacking in will-power even more than in intelligence, are sent out to work. Many go into domestic service, and in certain instances do well enough. These are, generally, in small middle-class households where the mistress does a large part of the work and takes on herself all the responsibility. The weak-minded servant suits her because she is cheap, and there can be little doubt that in many cases these poor girls are treated with considerable harshness. They are trying, of course. Some are hopelessly dull of intellect, others display a spasmodic, unbalanced sharpness which is even more difficult to deal with. A task seems to be learned to-day, yet is hopelessly, ridiculously, ill-done on the morrow; feverish industry alternates with determined idleness; there are vanities, sulkings, neglects, which even the most considerate mistress finds it hard to put up with. And the mistresses who employ these girls are often not considerate, but punish as if fully endowed with brains girls whom they can afford to hire only because they are not.

And yet this is not the worst. Trace the careers of many of those girls sent out from homes and institutions at the age of fourteen. At seventeen or eighteen we find them in the workhouse—too often in the maternity ward. "Gone wrong," "fallen," so they are labelled for life, poor girls whose weakness should have been protected from the dangers of this world, their very innocence making them the easier prey of selfishness and vice. And what of their children, inheriting, as they are almost certain to do, their mother's mental weakness, with who can tell what vicious tendencies from their father? The problem, being shirked and left unsolved, repeats itself perhaps in an aggravated form. The present writer knows one Roman Catholic orphanage (Smyllum, Lanarkshire) where the head of the institution, a devout nun, but a most practical woman, has added an elaborate steam laundry to the establishment chiefly because she found among her charges many girls who, though perfectly competent to work under proper supervision, were yet unable to be trusted to make their own way in the world. The laundry will probably pay in the end, for Sister Theresa is a good woman of business, and her work has many sympathizers among Protestants as well as Catholics; but, even

* "The Feeble-Minded Child and Adult: A Report on an Investigation of the Physical and Mental Condition of 50,000 School Children, with suggestions for the better Education and Care of the Feeble-minded Children and Adults." (London: Swan Sonnenschein.)

though it were to prove a pecuniary loss, it would none the less point out the true way of helping the feeble-minded.

They must work, for work may improve their intellect, and at least will exercise a soothing influence upon their sensitive, ill-balanced, nervous natures. But the work must be moderate in degree, carried out under pleasant surroundings, and should involve very little personal responsibility. Wherever such a plan has been tried — at Elberfeld, Christiania, Stockholm, Copenhagen — good results have followed. The pupils have proved themselves educable to a considerable degree, and have finally attained a profitable proficiency in various forms of manual labour. In England the Leicester School Board has led the way in forming a special class for those children who are not bright enough to profit by ordinary instruction. The experiment will be watched with interest, and, if successful, we do not doubt that it will be followed in many other places.

